# Mutation screening of patients with Alzheimer disease identifies *APP *locus duplication in a Swedish patient

**DOI:** 10.1186/1756-0500-4-476

**Published:** 2011-11-01

**Authors:** Håkan Thonberg, Marie Fallström, Jenny Björkström, Jacqueline Schoumans, Inger Nennesmo, Caroline Graff

**Affiliations:** 1 Genetics Unit, Dept of Geriatric Medicine, Karolinska University Hospital, Huddinge, Sweden; 2 Department of Molecular Medicine and Surgery and Center for Molecular Medicine, Karolinska Institutet, Stockholm, Sweden; 3 Cancer Cytogenetic Unit, University Hospital of Lausanne, Switzerland; 4 Department of Pathology, Karolinska University Hospital, Huddinge, Sweden; 5 Department of Neurobiology, Care Sciences and Society (NVS), Karolinska Institutet, KI-Alzheimer Disease Research Center, Stockholm, Sweden

## Abstract

**Background:**

Missense mutations in three different genes encoding amyloid-β precursor protein, presenilin 1 and presenilin 2 are recognized to cause familial early-onset Alzheimer disease. Also duplications of the amyloid precursor protein gene have been shown to cause the disease. At the Dept. of Geriatric Medicine, Karolinska University Hospital, Sweden, patients are referred for mutation screening for the identification of nucleotide variations and for determining copy-number of the *APP *locus.

**Methods:**

We combined the method of microsatellite marker genotyping with a quantitative real-time PCR analysis to detect duplications in patients with Alzheimer disease.

**Results:**

In 22 DNA samples from individuals diagnosed with clinical Alzheimer disease, we identified one patient carrying a duplication on chromosome 21 which included the *APP *locus. Further mapping of the chromosomal region by array-comparative genome hybridization showed that the duplication spanned a maximal region of 1.09 Mb.

**Conclusions:**

This is the first report of an *APP *duplication in a Swedish Alzheimer patient and describes the use of quantitative real-time PCR as a tool for determining copy-number of the *APP *locus.

## Background

Missense mutations in three different genes encoding amyloid-β precursor protein [1] (APP, [MIM 104760]), presenilin 1 [2] (PSEN1, [MIM 104311]) and presenilin 2 [3] (PSEN2, [MIM 600759]) are recognized to cause familial early-onset Alzheimer disease (EO-AD; [MIM 104300]). The mutations are known to functionally change the proteolytic processing of the APP protein, which leads to an increased Aβ42/Aβ40 ratio and increased Aβ-deposition in the brain. In addition to nucleotide variations, an increased dosage of the *APP *gene is also known to cause Alzheimer disease (AD) [4]. This is in agreement with previous observations that patients with Down syndrome (DS, trisomy 21, [MIM 190685]), who carry an extra copy of the *APP *gene, develop AD early in age [5-7]. Duplication of the *APP *locus has been detected in six unrelated French families [4,8], two Dutch families [9], one Finnish family [10], five families from a UK screen [11], and in two Japanese families [12]. The reported size of *APP *duplications range from 0.5 to 6.5 Mb, with the exception of an extended discontinuous duplication identified to be 15.5 Mb plus 1.5 Mb found in the UK screen [11]. Notably, a study on EO-AD cases from Finland and Sweden failed to identify duplications of the *APP *locus in a total of 141 AD patient, and concluded that the prevalence in these populations are low [13].

At the Dept. of Geriatric Medicine, Karolinska University Hospital, Sweden, patients are referred for mutation screening at the Genetic Unit for the identification of nucleotide variations and for determining copy-number of the *APP *locus. During the period between April 2008 and June 2010, a total number of 22 patients with clinical AD were referred for mutation screening, and the first finding of an *APP *duplication in a Swedish patient was made. The duplication was identified by analysis of microsatellite markers and quantitative real-time PCR, while confirmed by array-based comparative genome hybridization (aCGH). The duplication covers 1.09 Mb on chromosome 21q21, including the entire gene for *APP*. In this cohort of patients referred for mutation screening, the genomic duplication occurs at a frequency of 4.5% (1/22). This shows the importance of continued screening for *APP *locus duplication in Swedish AD patients, in parallel with sequencing efforts for the detection of nucleotide variations.

## Methods

### Patient and family history

Twenty-two patients were referred for mutation screening in the genes *APP*, *PSEN1*, *PSEN2 *at the Genetics Unit as part of the clinical investigation at the Dept of Geriatric Medicine, Karolinska University Hospital, Sweden. We made a subjective classification of the 22 subjects into four groups: early onset familial AD (EO-FAD); familial AD (FAD); possible familial AD (poss. FAD) and AD (Additional file 1). Ten cases were classified as "EO-FAD", where an autosomal dominant history of early onset (≤65 years of age) in at least three affected family members in two generations were found. Four of the cases were classified as "FAD", where there was at least three affected family members in two generations but where the onset in one of the family members was >65 years of age, and thus not fulfilling the criteria for early-onset. Six of the subjects were categorized as "possible FAD", since the number of identified family members with AD were not sufficient to definitively conclude the nature of inheritance. Finally, two patients were referred for mutation screening on the basis of autopsy-confirmed severe cerebral amyloid angiopathy (CAA) or because of an extreme early-onset, <30 years of age and classified as "AD".

The study was performed in accordance with the Helsinki Declaration, with informed consent and approval from the local ethics committee (Stockholm).

### DNA samples

Genomic DNA was extracted from peripheral blood using the manufacturer's protocol (Gentra Purgene Blood kit, Qiagen, Sweden). A DNA sample from an individual with trisomy 21 (T21) was used as a positive control for the microsatellite marker analyses and for the *APP *copy-number assay (Coriell Cell Repositories Camden, New Jersey, USA, (Catalog ID: GM02767).

### DNA sequencing

Sequencing reactions were performed using BigDye^® ^Terminator v3.1 Cycle sequencing kit (Applied Biosystems, Carlsbad, California, USA) followed by capillary electrophoresis on a ABI 3100 Genetic Analyzer (Applied Biosystems, Carlsbad, California, USA). Primers and PCR conditions are available on request.

### Microsatellite marker analysis

DNA from patients, D01-D22, were amplified with primers for microsatellite marker GDB:188463 (D21S265), located 330 kb centromeric to the *APP *gene, and for GBD:196999 (APP-dint), located in intron 1 of *APP *[4]. Fluorescent PCR-products were separated by capillary electrophoresis on an ABI 3100 instrument (Applied Biosystems, Carlsbad, California, USA) and the electropherograms were analyzed by using the GeneMapper 3.7 software (Applied Biosystems, Carlsbad, California, USA). The area under each allele's major peak was determined and the ratio for the two different allele-areas for a marker was calculated to evaluate copy-number variation. A balanced copy-number of two was scored for ratios between 0.8 and 1.4, whereas ratios less than 0.65 or greater than 1.8 were used to define samples with three alleles.

Amplification with Taq-polymerase can result in so-called stutter-peaks when two alleles only differ by two nucleotides in size [14]. Therefore, markers with alleles that differ by two nucleotides were allowed to have ratios between 1.4 - 1.8 and still score as a normal copy-number. Mono-allelic markers yielding only one peak were not informative for indicating copy-number.

### Copy-number assay

Quantitative real-time PCR was performed with the Taqman^® ^copy-number assay method according to the manufacturer's protocol (Applied Biosystems, Carlsbad, California, USA). The assays amplify target-regions within the *APP *locus in exon 1 (Assay ID: Hs00569527_cn), exon 7 (Assay ID: Hs02339796_cn), and in exon 17 (Assay ID: Hs00525904_cn). Additional assays amplify regions in closely located genes; *MIR155HG *(Assay ID: Hs04070445_cn), *ATP5J *(Assay ID: Hs02552822_cn), *CYYR1 *(Assay ID: Hs01271635_cn), and *ADAMTS1 *(Assay ID: Hs00609065_cn) (Additional file 2). Briefly, target assay-primers with a FAM-dye labeled probe and the calibrator RNase P-primers with a VIC-dye labeled probe, were used together in a duplex real-time PCR-reaction. The cycle threshold (Ct)-value at log-linear phase was used to calculate delta Ct-values_sample _(dCt_sample_) between the target assay and calibrator assay (Ct_Assay _- Ct_Calibrator = _dCt_sample_). The mean from tetraplicates of delta Ct_samples _from each sample DNA (D01-D22, and T21), was used for calculating relative copy-number normalized to the mean dCt-value of the trisomy 21 control DNA (T21) by the equation: 2^^(dCtT21-dCtsample)^*3.

### Comparative genome hybridisation

The commercially available 180 K oligonucleotide catalog design (design ID 022060, Agilent Technologies, Palo Alto, CA, USA) with complete genome coverage and overall median probe spacing of 13 kb was used. Experiments were performed according to the manufacturer's protocol with minor modifications. Briefly, patient DNA and a gender-matched control (Promega, Wisconsin, USA) were labeled by random priming (Enzo life sciences, New York, USA) with Cy3-dUTP and Cy5-dUTP respectively. After denaturing the probe and pre-annealing with 50 μg of Cot-1 DNA (Invitrogen, California, USA), hybridization was performed at 65 °C with rotation for 24 hours. The array was scanned with the Agilent microarray scanner (Palo Alto, CA, USA). Feature Extraction (v10.2) and DNA Analytics (v4.0) software packages (Agilent technologies) were used for analysis and duplication breakpoints were determined by visual inspection of the numerical normalized log_2 _ratio values in the table-view of the DNA analytics software package. The genomic positions refer to the Human Genome Build 36.1 (UCSC Genome Browser, assembly March2006).

## Results

Twenty-two patients were referred for mutation screening in the genes *APP*, *PSEN1 *and *PSEN2 *during the period of April 2008 to June 2010 at the Dept. of Geriatric Medicine, Karolinska University Hospital, Sweden. The screening involved genomic sequencing of exons 16-17 in *APP*, exons 2-12 in *PSEN1*, exons 3-7 and exon 12 in *PSEN2*. No variation in *APP *was detected in any of the 22 DNA samples. In contrast we identified polymorphic SNPs in both *PSEN1 *and *PSEN2*. Polymorphic SNPs in *PSEN2 *were frequently detected and, in addition to the seven previously reported polymorphisms, we also found a novel variation at IVS6 +91 (G>C; GRCh37:1:227075950) (see Additional file 3 and Additional file 4). The functional implication on pathogenesis was tested by *in silico *methods. We were not able to detect a functional impact of the variation, neither by splice-prediction analysis nor by examining conservation between species (Additional file 4). Taken together, our sequencing effort did not find variations that could explain the diseased state of the patients.

**Figure 1:**
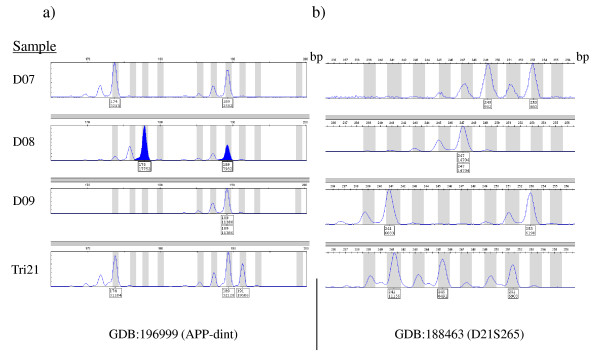
**Electropherograms of microsatellite markers for three different DNA, (D07-D09), and for trisomy 21-DNA control (T21)**. On the *y*-axis is peak height and on the *x*-axis is the marker size in base-pairs. Marker alleles show up as major peaks and indicated in boxes underneath each allele are size in base-pair and the peak-area. a) Four individual electropherograms showing representative amplification of marker GDB:196999 (APP-dint). The blue peaks, for alleles 178 and 189, show the suspected copy-number variation in DNA sample D08. b) Four individual electrographs showing representative amplification of marker GDB:188463 (D21S265).

To address the possibility of a duplication of *APP*, two microsatellite markers GDB:196999, (APP-dint), and GDB:188463, (D21S265) were PCR amplified with fluorescent primers. When analyzing the 22 DNA samples, 16 were heterozygous (polymorphic) for the marker GDB:196999 and 20 were heterozygous for marker GDB:188463. In our set-up, the size of alleles for marker GDB:196999 ranged from 163 bp to 191 bp, and the size of alleles for GDB:188463 ranged from 241 bp to 255 bp (Table 1). The allele-ratio calculated for marker GDB:196999 was between 0.8 - 1.4 indicating a balanced diploid copy-number, except for sample D08 (Table 1). In sample D08, the areas for alleles 174 bp and 189 bp for GDB:196999 had a ratio of 2.24 (17752/7932) indicating an unbalanced 2:1 copy-number (Figure 1). Six of the individuals were monoallelic for the marker. When calculating the allele-ratios for marker GDB:188463 in the sample-set, thirteen out of 22 DNA samples had ratios between 0.8 - 1.4. Seven samples had ratios between 1.4 - 1.8 and five of these samples had alleles that only differed by two nucleotides in size (D03, D18, D20 - 22); thereby leading to inflation of the area of the shorter allele (Table 1). The ratios for D13 and D15 were slightly higher than 1.4 and monoallelic for GDB:196999. Two samples were monoallelic for marker GDB:188463 (D08 and D16) and therefore did not generate any allele ratios. As an example, in Figure 1, DNA sample D07 carries two alleles for both markers, GDB:196999 and GDB:188463, and the calculated ratio for alleles GDB:196999-174 and GDB:196999-189 was 1.24, (3213/2582), indicating a balanced copy-number of two. Similarly, the ratio for the alleles of GDB:188463 is calculated to be 1.02 (902/883) for sample D07. Also illustrated, sample D08 was not informative for marker GDB:188463 since it only carried one allele, the 247 bp (Figure 1b), but an unbalanced copy-number are visible as the ratio, 2.24 (17752/7932), for the alleles of marker GDB:196999. Furthermore, sample D09 was not informative for marker GDB:196999 since it only carried one allele (189 bp), but could be examined at the GDB:188463 marker, with a ratio of 1.08 (6683/6190) (Figure 1b). The control DNA carrying trisomy 21 displayed three alleles for both examined markers (Figure 1a-b). The finding indicated that sample D08 carries a duplication of the genomic locus for marker GDB:196999.

**Table 1:** Copy-number determination in 22 AD patients referred for mutation screening.

	GDB:196999	(APPdint)	GDB:188463	(D21S265)	CNA-APP		
	alleles	ratio	alleles	ratio	E1	E7	E17
**D01**	185/189	1,34	243/253	1,03	2,17	2,01	2,18
**D02**	174/189	1,19	241/245	1,37	2,11	1,91	2,17
**D03**	174/189	1,18	245/247	1,57	1,93	1,87	2,07
**D04**	189/199	1,11	245/247	1,18	2,14	1,90	2,09
**D05**	174/189	1,27	245/249	0,92	2,16	2,01	2,05
**D06**	191	-	243/249	0,93	2,28	2,33	2,17
**D07**	174/189	1,24	249/253	1,02	2,00	2,04	2,08
**D08**	174/189	**2,24**	247	-	**3,42**	**3,49**	**3,44**
**D09**	189	-	241/253	1,07	2,03	2,06	2,12
**D10**	163/174	1,21	245/255	1,04	1,67	1,81	1,92
**D11**	174/189	1,00	247/255	1,19	1,78	2,23	1,92
**D12**	163/174	1,18	249/255	0,97	1,65	1,71	1,85
**D13**	189	-	247/251	1,45	1,90	1,83	2,06
**D14**	174/178	1,35	245/249	1,38	1,74	1,55	1,78
**D15**	189	-	249/253	1,53	2,15	2,01	1,95
**D16**	163/189	1,30	247	-	nd	nd	nd
**D17**	189	-	241/247	1,24	1,52	1,63	1,71
**D18**	174/191	1,21	247/249	1,63	1,54	1,81	1,79
**D19**	178/189	1,33	247/255	1,12	1,54	1,58	1,63
**D20**	178/189	0,95	247/249	1,67	1,93	2,27	2,18
**D21**	189	-	247/249	1,73	1,92	2,22	2,30
**D22**	189/193	1,36	247/249	1,70	2,02	2,02	2,00

DNA samples are numbered D01-D22. From left; microsatellite-analysis for marker GDB:196999 (APP-dint) and GDB:188463 (D21S265). The column "alleles" for each of the two markers gives the allele size in base-pairs. Also, for the two markers, and given in the column "ratio", are the ratios between the peak-areas under each alleles major peak. Relative copy-number values from the copy-number assays (CNA-APP) targeting APP-exon 1 (E1), exon 7 (E7) and exon 17 (E17). The values in each column are calculated as relative to trisomy 21-DNA control and given as mean of tetraplicates. The abbreviation "nd" stands for not determined. Bold figures indicate ratio and copy-number of samples with suspected copy-number variation.

**Figure 2:**
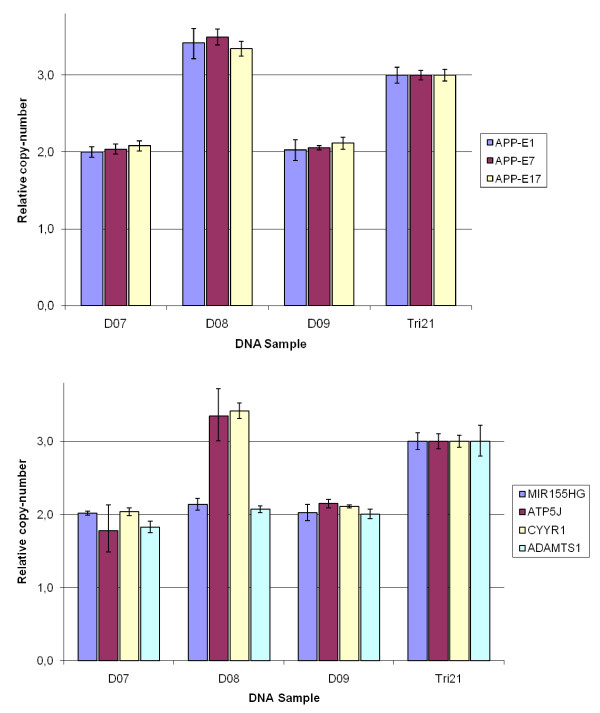
**Bar graph showing copy-number from the copy-number assay targeting *APP *exons (top) and genes flanking *APP *gene (lower)**. On the *y*-axis is the copy-number calculated relative to trisomy 21-DNA control, and on the *x*-axis are three patient DNA samples, D07, D08, D09 and the trisomy-21 DNA (Tri21). For top graph, three different assays at the *APP *locus were used, positioned in Exon1, (blue), Exon7 (red), and in Exon17 (yellow).). For lower graph, four different assays were used targeting the genes of; *MIR155HG*, (blue), *ATP5J *(red), *CYYR1 *(yellow), and *ADAMTS1 *(light blue). Assays were performed in tetraplicate and bars are shown as mean ±standard deviation.

To confirm the *APP *copy-number detected by microsatellite analysis, we used a quantitative real-time PCR method (The TaqMan Copy Number Assay, Applied Biosystems Carlsbad, California). The number of copies was determined by comparing patient DNA with a positive control DNA from a subject with trisomy 21 (copy-number of three). Three different exons of the *APP *gene were amplified by a duplex real-time PCR method for exon 1, exon 7, and exon 17. By calculating the relative copy-number, samples D07 and D09 were found to be diploid for all three amplicons (Figure 2, top). In contrast, sample D08 was found to have a copy-number value of three for all three amplicons. Thus, the copy-number assay confirmed the presence of three copies detected by the microsatellite analysis for D08. All the other DNA samples were determined by the copy-number assay to have relative copy-number values of 1.52-2.33, indicating a copy-number of two (Table 1). Noteworthy, the copy-number assay for samples D13 and D15 showed no variation in any of the exons examined and thus could be determined to carry normal number of the *APP *gene.

We wanted to address how far the duplication extended into the flanking chromosomal regions and if any neighboring genes were duplicated in addition to *APP*. For this purpose, copy-number assays targeting the genes *MIR155HG*, *ATP5J*, *CYYR1*, and *ADAMTS1 *were analyzed (for locations see Additional file 2). The relative copy-number for samples D07 and D09 was two for all of the four investigated genes (Figure 2 lower). The relative copy-number values ranged from 1.78 (*ATP5J*, sample D07) to 2.15 (*ATP5J*, sample D09). The duplication established in sample D08 was determined to include the locus for the *ATP5J *gene located centromeric as well as the locus for the *CYYR1 *gene located telomeric of *APP *and the relative copy-number for these two assays were close to three, (3.34 and 3.41 respectively) (Figure 2 lower). However, the duplication did not include the more distal gene *MIR155HG*, located centromeric, or the gene for *ADAMTS1 *located telomeric (Figure 2 lower) as indicated by the relative copy-number values of 2.14 and 2.07 respectively. We could therefore limit the duplication to a maximal region of 1.27 Mb on chromosome 21 between genomic position 25 868029 bp and 27 138783 bp (Build 36.1).

**Figure 3:**
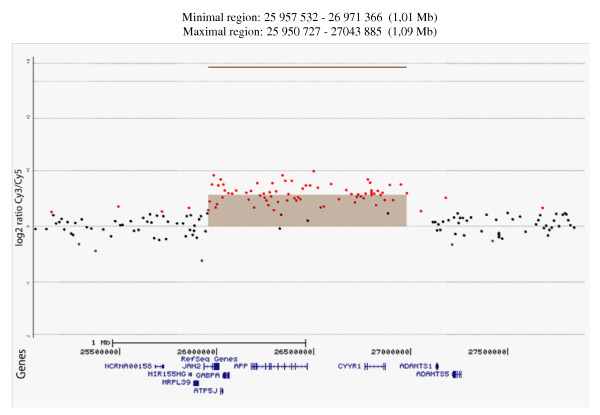
**Plot from array-CGH made with DNA analytics software**. On the *y*-axis is the log^2 ^ratio signal intensity between Cy3-labeled sample D08 and Cy5-labeled diploid control DNA. The *x*-axis shows the genomic position of annotated genes (Build 36.1). The colored dots indicate the signal intensity from hybridization of the patient DNA D08 compared to the signal of the control sample. Equal signal is shown as black dots, a stronger signal as red dots, and green dots when the signal is weaker from hybridizing with the patient DNA D08. Illustrated as a brown box is the construction of the maximal region between coordinates 25 950727 - 27 043885 that is duplicated in patient sample D08. Coordinates for both maximal and minimal region of duplication as determined by array-CGH are given above the plot.

To fine-map the duplicated region, array-CGH was used by hybridizing DNA sample D08 to a 180 k array covering the *APP *locus. The method compares hybridization signals from the D08 DNA with a diploid control DNA. The signal intensities were plotted against positions on the chromosome and shows that the duplication covers a region on chromosome 21 restricted to a maximum size of 1.09 Mb within coordinates 25 950727 bp - 27 043885 bp, and to a minimum size of 1.01 Mb within coordinates 25 957532 bp - 26 971 366 bp (Figure 3). Thus, the array-CGH confirms the presence of a genomic duplication including the *APP *gene in sample D08.

## Discussion

The genetic causes for EO-FAD are strongly linked to three genes: *APP, PSEN1*, and *PSEN2*. The frequency in which mutations in these genes contribute to EO-FAD is reported to range from 17% to around 80% [15-18], depending on the population examined, the sample size, and the diagnostic criteria (for review see Avramopouls 2009) [19]. At the Dept. of Geriatric Medicine, Karolinska University Hospital, Sweden, patients with a suspected genetic cause of AD are referred for mutation screening in the genes *APP, PSEN1*, and *PSEN2*. In addition to using sequencing for mutation screening, we have developed a protocol for analyzing the copy-number of *APP*. The protocol includes both a microsatellite analysis and a copy-number assay. Here we report on the results from sequence (see Additional file 3 and Additional file 4) and copy-number analyses of 22 AD patients referred for mutation screening on the basis of their familial history with the detection of an *APP *duplication in one of the 22 patients. The calculated duplication frequency found in the present study depends on the number of samples considered, i.e. when only the 10 EO-FAD patients are included the frequency is 10%, and when including all 22 samples the frequency is 4.5%.

In contrast, our sequencing efforts did not identify any nucleotide variations in the three genes that could explain the diseased state. The low frequency of mutations in the Swedish population is also notable in earlier studies [20,21]. The mutations found so far in Swedish patients are six in total, and includes two in *APP *[22,23] and four in *PSEN1 *[20,24,25]. This finding strengthens the notion that mutations in *APP*, *PSEN1 *and *PSEN2 *are less frequent in Swedish families than in the reports on European EO-FAD. Significantly, 10 of the 22 patients had a family history with early-onset (≤ 65 year-of age), and an additional four of the patients came from families with an autosomal dominant history of AD.

The recognition of *APP *duplication as a genetic cause for AD has raised the issue of to what degree it explains familial EO-AD. Others have reported frequencies of *APP*-duplications between 2.7% and 8% in familial cases of AD [4,9,12]. This indicates that the contribution of *APP *duplications as a cause for the disease is one third to half of that from *APP *nucleotide missense mutations [15]. A report on screening for *APP *duplication in 77 Swedish and 64 Finnish patients did not succeed in finding any copy-number variations [13]. Again, this indicated that frequencies of genetic variations may differ between populations of different origin. However, in our study, the frequency of duplications is in the range of previous reports, with frequencies of 8% in French [4] and Japanese AD-families [12], and at 2.7% in Dutch familial AD-cases [9].

**Figure 4:**
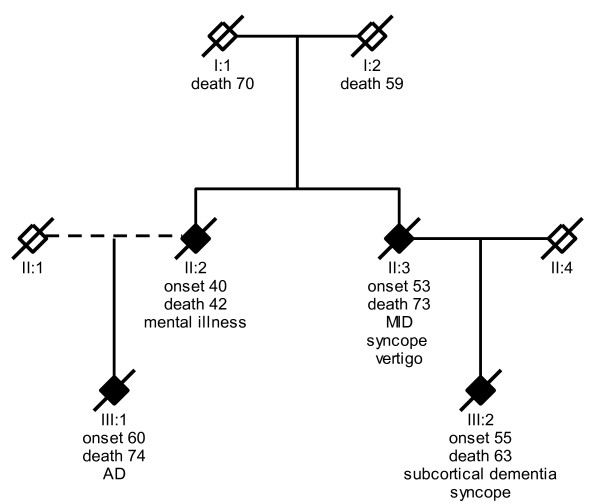
**Pedigree from the duplication family to which patient D08 (III:1) belongs**. Black box indicates dementia diagnosis, crossed box indicates deceased individual. Given below family members are age of onset, age of death, and clinical observations. Abbreviations used are AD for Alzheimer disease, and MID for multi-infarct dementia.

The duplication at chromosome 21 we reported is restricted to a maximum size of 1.09 Mb and to a minimum size of 1.01 Mb, which includes the genes for *MRLP39*, *JAM2*, *ATP5J*, *GABPA*, *APP*, and *CYYR1*. All but two of the reported APP-duplications are of different sizes, and thereby include different genes. From the reports of different-sized duplications, it has also been suggested that there is no single genomic architectural feature that serves as a recombination substrate, but that the *APP *locus may be a hotspot region for recombination due to multiple low copy repeats [9]. This is supported by our finding in a Swedish patient, since it adds a novel-sized duplication. It has been suggested that sequencing of the duplication break-points may shed further light on the molecular basis of the recombination in the region. The two different-sized duplications found in Dutch families contain only the *APP *gene which strongly suggests that *APP *duplication is the only requirement for causing AD. The clinical characteristics in patient with APP-duplications are typically early-onset dementia, cerebral amyloid angiopathy (CAA) and intracerebral hemorrhage (ICH). When looking within families there are phenotypes of seizure and dementia with Lewy-bodies [8-10,26], but clearly these are not found in all diseased individuals. Rather, intra-familial diversity is commonly found. In the Swedish family originating from proband III:2, (DNA sample D08), there was an early onset at 55 years in the cousin (III:4) (Figure 4). Individual III:4 had a medical history of syncope and a diagnosis of subcortical dementia. Similarly, the history of individual II:5 also contained remarks of syncope, as well as vertigo and the diagnosis of multi-infarct dementia. Individual II:4 had a history of mental illness and was hospitalized under the diagnosis of schizophrenia and psychosis. The duplication has only been confirmed in proband III:2, since there are no available DNA samples from other affected family members.

## Conclusions

To conclude, we report the first Swedish EO-AD patient carrying an *APP*-duplication. We have successfully used the copy-number assay, showing that it is reliable and straightforward to use. It is also a good complement to the use of microsatellites to determine copy-number since these require the presence of heterozygous markers to be informative. We would like to emphasize the value of continued screening for *APP-*duplications in the Swedish population of EO-AD, since we found it to be more frequent than nucleotide sequence variations in our cohort of patients referred for genetic mutation screening.

## Competing interests

The authors declare that they have no competing interests.

## Authors' contributions

HT carried out the molecular genetic studies and made the *in-silico *analysis. JS carried out the genome hybridization study. MF and JB carried out the genealogy of the patients as genetic counselors. IN judged clinical data and carried out neuropathology. CG was responsible for referral of patients to mutation screening and conceived the study. HT drafted the manuscript and HT, JS, MF, JB, IN, and CG contributed to writing the manuscript. All authors read and approved the manuscript.

## Supplementary Material

Additional file 1 **Table showing classification of the 22 cases of AD according to their family history with table legend**. Click here for file

Additional file 2 **Schematic picture of the *APP *locus with surrounding genes with figure legend**. Click here for file

Additional file 3 **Table showing nucleotide variations found in the 22 DNA samples for sequencing of *APP*, *PSEN1*, and *PSEN2 *with table legend**. Click here for file

Additional file 4 **Method and result of on *In-silico *analysis of nucleotide variation IVS6 +9 and**. Click here for file
