# Supplementary material for: Mutation screening of patients with Alzheimer disease identifies APP locus duplication in a Swedish patient
Source: BMC Res Notes. 2011 Nov 1;4:476. doi: 10.1186/1756-0500-4-476 (PMC3216298; doi:10.1186/1756-0500-4-476)
Supplement: Additional file 1 — Table showing classification of the 22 cases of AD according to their family history with table legend. [file 1756-0500-4-476-S1.PDF]

Additional file 1

DNA sample	Classed as;	Patient onset	Neuropath	# affected	Onset, family member(s)	Neuropath
**D01**	EO-FAD	55	defAD, Braak V-VI	4	64, 65, 67	
**D02**	FAD	50	defAD, Braak VI, CAA	3	65, > 75	
**D03**	poss FAD	70		2	55	
**D04**	poss FAD	59		2	61*	defAD
**D05**	FAD	64		4	62, 68, > 76	
**D06**	poss FAD	45		2	76	
**D07**	poss FAD	56		2	< 70	
**D08**	EO-FAD	60		4	40, 55, < 60	
**D09**	EO-FAD	56		3	62*, 65	defAD, Braak VI, CAA
**D10**	EO-FAD	55		7	55*, 57, 60, 62, 65, 74	defAD, Braak V-VI, CAA
**D11**	EO-FAD	60		9	< 60*, < 60, 61, 62, < 65, 78, < 80, <80	brain atrophy
**D12**	EO-FAD	<60	defAD, Braak V-VI, CAA	4	61, 65*, 68	defAD, Braak V-VI
**D13**	poss FAD	62	defAD, Braak V-VI, CAA	2	62	
**D14**	FAD	65		4	62, unknown, 82	
**D15**	EO-FAD	65		3	50, 60	
**D16**	AD	68	severe CAA, severe tauopathy	1	unknown	
**D17**	EO-FAD	<55		3	< 65, < 65	
**D18**	FAD	68		4	63, 68*, 76	defAD, Braak V-VI, CAA
**D19**	poss FAD	66		2	62	
**D20**	EO-FAD	< 65		4	< 60, < 60, < 60	
**D21**	AD	<30		1	unknown	
**D22**	EO-FAD	59	defAD, Braak V, CAA	3	62, 65	

- Classification of the 22 cases of AD according to their family history of disease.

The patients age of onset, their neuropathology and the total number of sick family members are assessed together with the family members age of onset and their neuropathology to subjectively classify the cases into; EO-FAD, FAD, possible FAD, or AD. Definite AD (DefAD) is in accordance to CERAD criteria for age-related semiquantitative assessment of neuritic plaques. The abbreviation CAA is used for cerebral amyloid angiopathy scored by congophilic staining in the vessel walls of the brain and the Braak V-VI indicate neurofibrillary tangle staging according to Braak. Onsets indicated with asterisk (*) shows family member with neuropathology remark.
