# Supplementary material for: Mutation screening of patients with Alzheimer disease identifies APP locus duplication in a Swedish patient
Source: BMC Res Notes. 2011 Nov 1;4:476. doi: 10.1186/1756-0500-4-476 (PMC3216298; doi:10.1186/1756-0500-4-476)
Supplement: Additional file 2 — Schematic picture of the APP locus with surrounding genes with figure legend. [file 1756-0500-4-476-S2.PDF]

Additional file 2

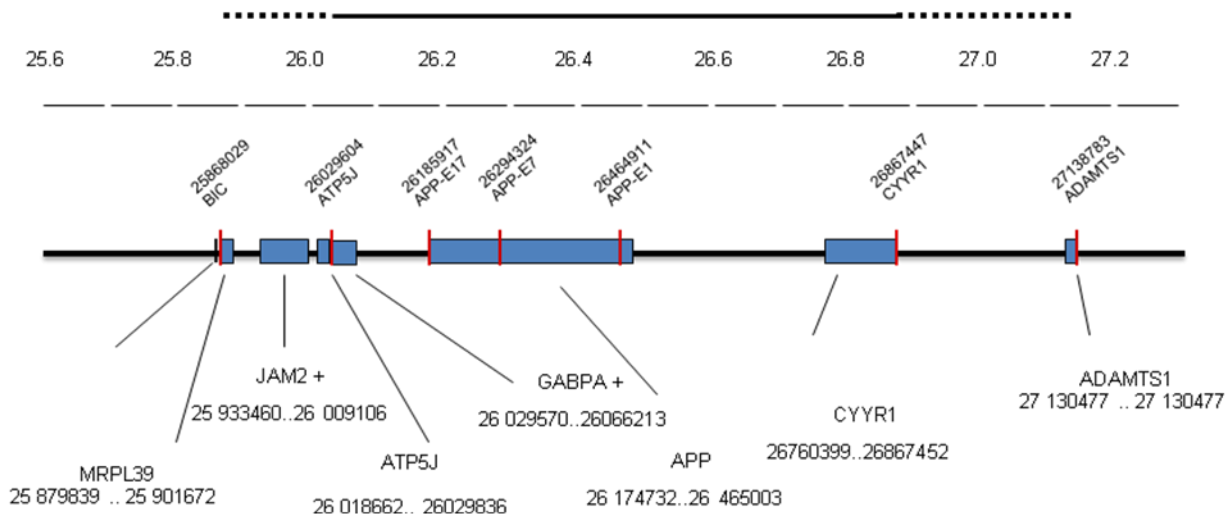

- Schematic picture of the *APP* locus with surrounding genes.

Indicated on top as unbroken line is the minimal region duplicated in sample D08, and as a dotted line is the maximal region. Light blue box show genes annotated to the region and red lines show the positions of copy-number assays used. Figures are chromosomal positions in base-pair from Human Genome Build 36.1.
