# Supplementary material for: Mutation screening of patients with Alzheimer disease identifies APP locus duplication in a Swedish patient
Source: BMC Res Notes. 2011 Nov 1;4:476. doi: 10.1186/1756-0500-4-476 (PMC3216298; doi:10.1186/1756-0500-4-476)
Supplement: Additional file 3 — Table showing nucleotide variations found in the 22 DNA samples for sequencing of APP, PSEN1, and PSEN2 with table legend. [file 1756-0500-4-476-S3.PDF]

Additional file 3

	APP	PSEN1		PSEN2							
		rs165932	rs17125721	rs6759	rs11405	rs1046240	rs1295643	rs1295644	rs2236910	rs1800680	IVS6 +91
**D01**	-	G G	A A	C C	C T	C C	G G	C T	C G	G G	G G
**D02**	-	G T	A A	C T	C C	C T	A G	C C	C C	G G	G G
**D03**	-	G T	A A	T T	C C	T T	A A	C C	C C	G G	G G
**D04**	-	G G	A A	C T	C T	C T	A G	C T	C G	G G	G G
**D05**	-	G T	A A	C T	C C	C T	A G	C C	C C	G G	G G
**D06**	-	G T	A A	C C	C T	C C	G G	C T	C G	G G	G G
**D07**	-	T T	A A	C T	C T	C T	A G	C T	C G	G G	G G
**D08**	-	G G	A A	C C	C T	C C	G G	C T	C G	G G	G G
**D09**	-	T T	A A	C T	C C	C T	A G	C C	C C	G G	G G
**D10**	-	G T	A A	C T	C T	C T	A G	C T	C G	G G	G G
**D11**	-	G T	A A	C T	C C	C T	A G	C C	C C	G G	G G
**D12**	-	G T	A A	C T	C T	C T	A G	C T	C G	G G	G G
**D13**	-	T T	A A	T T	C C	T T	A A	C C	C C	G G	G G
**D14**	-	G T	A G	C C	C T	C C	G G	C T	C G	G G	C G
**D15**	-	G G	A A	C T	C T	C T	A G	C T	C G	G G	G G
**D16**	-	not seq.	not seq.	not seq.	not seq.	not seq.	not seq.	not seq.	not seq.	not seq.	not seq.
**D17**	-	G G	A A	T T	C C	T T	A A	C C	C C	G G	G G
**D18**	-	G T	A A	C C	C C	C C	G G	C C	C C	G G	G G
**D19**	-	T T	A A	C T	C C	C T	A G	C C	C C	G G	G G
**D20**	-	T T	A A	C T	C C	C T	A G	C C	C C	A G	G G
**D21**	-	G T	A A	T T	C C	T T	A A	C C	C C	A G	G G
**D22**	-	T T	A A	C T	C C	C T	A G	C C	C C	G G	G G
MAF		0,476	0,024	0,476	0,214	0,476	0,476	0,214	0,214	0,048	0,024

- **Nucleotide variations found in the 22 DNA samples when subjected to sequencing of *APP*, *PSEN1* and *PSEN2*.** Minor allele frequency (MAF) given at the bottom of the table are calculated from the 22 samples within this study. No variations were found in *APP* indicated by dash (-).
