# Supplementary material for: Mutation screening of patients with Alzheimer disease identifies APP locus duplication in a Swedish patient
Source: BMC Res Notes. 2011 Nov 1;4:476. doi: 10.1186/1756-0500-4-476 (PMC3216298; doi:10.1186/1756-0500-4-476)
Supplement: Additional file 4 — Method and result of on In-silico analysis of nucleotide variation IVS6 +9 and. [file 1756-0500-4-476-S4.PDF]

Supplement methods and result

***In-silico* analysis:**

The nucleotide variation IVS6 +91 (G > C) for *PSEN2* identified in sample D14 was tested for impact on splicing by two on-line tools, SpliceView, (http://bioinfo.itb.cnr.it/oriel/splice-view.html) [S2], and with NetGene2, (www.cbs.dtu.dk/services/NetGene2) [S2]. The sequence analyzed for prediction of splicesite was GRCh37:1: 227075364-227076864. We also made comparative genomic alignment for the sequence GRCh37:1: 227075880 bp – 227075955 bp against 12 eutherian mammals at the website www.ensembl.org.

Result

The result of genomic sequencing of exons 16-17 in *APP*, exons 2-12 in *PSEN1*, exons 3-7 and exon 12 in *PSEN2* are summarized in TableS2. No variation in *APP* was detected in any of the 22 DNA samples. Polymorphic SNP rs165932 and rs1712752 in *PSEN1* were detected with minor-allele frequency, (MAF), of 0,476 and 0,024 respectively. Eight different variations were detected in PSEN2 with their MAF indicated in Table S2. Seven of theses variations were previously reported SNP´s, whereas one, IVS6 +91, had not been reported before (Table S2).

No change in prediction of splicing could be seen after analyzing the variation PSEN2 IVS6 +91 *in-silico* by the use of the tools SpliceView, and NetGene2. Aligning the region which comprises the variation, demonstrated that the nucleotide G was conserved in 5 primates, whereas in the other 6 mammals, the region was either missing or the conservation did not include that particular nucleotide. Summarized, we were not able to link the variation seen in sample D14 at *PSEN2* IVS6 +91 with functional implications for splicing and no conservation throughout species was found.

and in *PSEN2*. Polymorphic SNPs in *PSEN2* were frequently detected and, in addition to 7 previously reported polymorphisms, we also found a novel variation at IVS6 +91 (G>C; GRCh37:1:227075950) (see Supplement and Table S2).

Supplement references

S2. Hebsgaard SM, Korning PG, Tolstrup N, Engelbrecht J, Rouze P, Brunak S: **Splice site prediction in Arabidopsis thaliana pre-mRNA by combining local and global sequence information**. *Nucleic Acids Res* 1996, **24**(17):3439-3452.

S2. Rogozin IB, Milanesi L: **Analysis of donor splice sites in different eukaryotic organisms**. *J Mol Evol* 1997, **45**(1):50-59.
